# A Nine-Strain Bacterial Consortium Improves Portal Hypertension and Insulin Signaling and Delays NAFLD Progression In Vivo

**DOI:** 10.3390/biomedicines10051191

**Published:** 2022-05-20

**Authors:** Iris Pinheiro, Aurora Barberá, Imma Raurell, Federico Estrella, Marcel de Leeuw, Selin Bolca, Davide Gottardi, Nigel Horscroft, Sam Possemiers, María Teresa Salcedo, Joan Genescà, María Martell, Salvador Augustin

**Affiliations:** 1 MRM Health NV, 9052 Ghent, Belgium; marcel.deleeuw@mrmhealth.com (M.d.L.); selin.bolca@mrmhealth.com (S.B.); davide.gottardi2@unibo.it (D.G.); nigel.horscroft@mrmhealth.com (N.H.); sam.possemiers@mrmhealth.com (S.P.); 2 Liver Unit, Department of Internal Medicine, Hospital Universitari Vall d’Hebron, Institut de Recerca Vall d’Hebron, Universitat Autònoma de Barcelona, 08035 Barcelona, Spain; aurora.barbera@vhir.org (A.B.); imma.raurell@vhir.org (I.R.); dr.estrella1@gmail.com (F.E.); jgenesca@vhebron.net (J.G.); maria.martell@vhir.org (M.M.); 3 Centro De Investigación Biomédica en Red de Enfermedades Hepáticas y Digestivas, Instituto De Salud Carlos III, 28029 Madrid, Spain; 4 Pathology Department, Hospital Universitari Vall d’Hebron, Universitat Autònoma de Barcelona, 08035 Barcelona, Spain; mtsalcedo@vhebron.net

**Keywords:** NAFLD, gut microbiome, bacterial consortium, portal hypertension, fibrosis

## Abstract

The gut microbiome has a recognized role in Non-alcoholic fatty liver disease (NAFLD) and associated comorbidities such as Type-2 diabetes and obesity. Stool transplantation has been shown to improve disease by restoring endothelial function and insulin signaling. However, more patient-friendly treatments are required. The present study aimed to test the effect of a defined bacterial consortium of nine gut commensal strains in two in vivo rodent models of Non-alcoholic steatohepatitis (NASH): a rat model of NASH and portal hypertension (PHT), and the Stelic animal (mouse) model (STAM™). In both studies the consortium was administered orally q.d. after disease induction. In the NASH rats, the consortium was administered for 2 weeks and compared to stool transplant. In the STAM™ study administration was performed for 4 weeks, and the effects compared to vehicle or Telmisartan at the stage of NASH/early fibrosis. A second group of animals was followed for another 3 weeks to assess later-stage fibrosis. In the NASH rats, an improvement in PHT and endothelial function was observed. Gut microbial compositional changes also revealed that the consortium achieved a more defined and richer replacement of the gut microbiome than stool transplantation. Moreover, liver transcriptomics suggested a beneficial modulation of pro-fibrogenic pathways. An improvement in liver fibrosis was then confirmed in the STAM™ study. In this study, the bacterial consortium improved the NAFLD activity score, consistent with a decrease in steatosis and ballooning. Serum cytokeratin-18 levels were also reduced. Therefore, administration of a specific bacterial consortium of defined composition can ameliorate NASH, PHT, and fibrosis, and delay disease progression.

## 1. Introduction

Non-alcoholic fatty liver disease (NAFLD), and its progressive form non-alcoholic steatohepatitis (NASH), is the hepatic manifestation of metabolic syndrome, and common comorbidities include obesity, type 2 diabetes mellitus (T2DM), hyperlipidemia, and hypertension [1]. Metabolic dysregulation is a hallmark of NAFLD, and insulin resistance (IR), hyperlipidemia, or dyslipidemia are common features. The gut microbiome has gained much attention in the past few years and its association with metabolic syndrome-related disorders, namely NAFLD, is well documented [2,3,4]. In this regard, numerous gut-born metabolites, synthetized by microbes, have been implicated in disease progression [4,5]. However, some gut-born metabolites may be of benefit to metabolic diseases. For instance, short-chain fatty acids (SCFAs), such as butyrate and propionate, have demonstrated positive effects, such as promoting the production of the anorexigenic peptides Peptide YY (PYY) and Glucagon-like peptide 1 (GLP1) [6]. Moreover, butyrate and propionate have well-established barrier protective and anti-inflammatory roles in the intestine [7]. Conversely, colonic inflammation lowers the abundance of SCFA-producing bacteria: a reduction in the genus *Faecalibacterium*, containing butyrate producers, has been observed in NASH patients [8]. Therefore, the growing interest in the gut–liver axis has opened avenues for the treatment or prevention of NAFLD using gut microbial approaches [9,10]. Previous studies have shown that the transfer of fecal microbiota collected from lean rats into NASH rats fed a high-fat glucose/fructose diet (HFGFD) restored the sensitivity to insulin of the hepatic protein kinase B (Akt)-dependent endothelial nitric oxide synthase (eNOS) signaling pathway, and thereby led to an improvement in intrahepatic vascular resistance (IHVR) and portal pressure (PP) [11]. Despite the observed protective effects on liver endothelial function, fecal transfer is unlikely to become a long-term therapeutic option, especially considering the chronic and progressive nature of this disease. Consequently, microbial-based therapies of defined composition would represent a more patient-friendly approach.

Therefore, in the current study we tested the efficacy of a bacterial consortium composed of nine human gut commensal strains capable of producing both butyrate and propionate, on a rat NASH model of portal hypertension (PHT) and compared it to that of fecal microbiota transplantation (FMT) from lean rats. We also tested the same nine-strain bacterial consortium in a rodent model described to develop liver fibrosis at histopathology: the STAM™ mouse model.

## 2. Materials and Methods

### 2.1. Rat NASH Model of Portal Hypertension (PHT)

**Figure 1:**
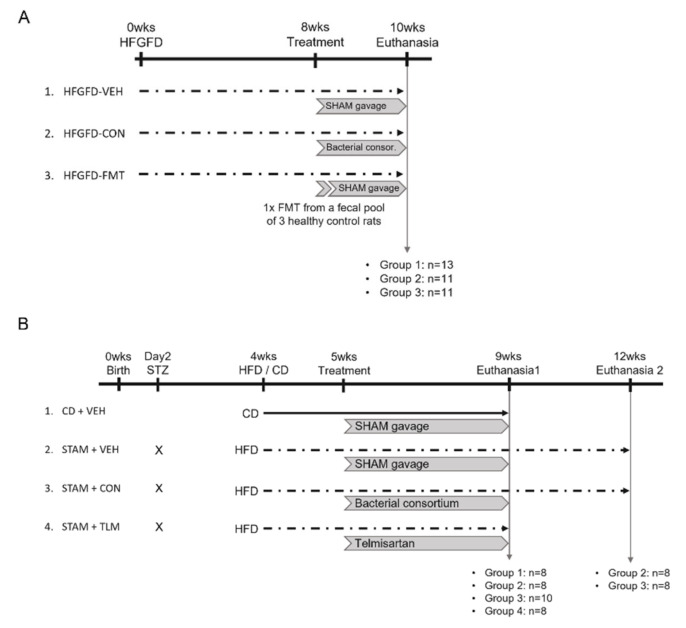
Experimental design. (**A**) Rat NASH model of PHT. Rats were fed a high-fat, high-glucose/fructose diet (HFGFD) for 8 weeks, after which they received either sham or bacterial consortium treatment for 2 additional weeks. HFGFD-VEH: group of NASH rats receiving sham gavage (vehicle); HFGFD-CON: group of NASH rats receiving the 9-strain bacterial consortium daily (2 × 10^9^ CFU/d, total counts); HFGFD-FMT: group of NASH rats receiving fecal microbiota transplantation from control lean rats (1× transplantation followed by sham gavage). (**B**) STAM™ mouse study. C57BL/6J male mice were subcutaneously injected with 200 µg STZ, a β-cell toxin, two days after birth. At 4 weeks of age animals received a high-fat diet (HFD) or regular control diet (CD). At 5 weeks of age treatment was initiated: control diet animals and STAM mice received sham gavage (CD + VEH and STAM + VEH, respectively) for a total of 4 weeks. In the two test groups, STAM animals received either the 9-strain consortium (10^9^ CFU/d, total counts) (STAM + CON) or Telmisartan (10 mg/kg/d) (STAM + TLM) also for a total of 4 weeks. At 9 weeks of age, the animals were sacrificed (euthanasia 1). Two groups of STAM animals belonging to the vehicle control or the consortium groups were followed for an additional three weeks until sacrifice at 12 weeks of age (euthanasia 2).

Male Sprague-Dawley OFA rats (Charles River Laboratories, l’Arbresle, France), weighting 200–220 g were used for the diet-induced NASH model, as previously described [11]. Briefly, animals had ad libitum access to a high-fat high-glucose/fructose diet (HFGFD) for 8 weeks (Figure 1A and Supplementary Materials), during which body weight and food and drink consumption were monitored weekly. After that, they were randomized into three groups: HFGFD-vehicle (HFGFD-VEH, *n* = 13), HFGFD-consortium of nine human commensal bacterial strains (HFGFD-CON, *n* = 11), and HFGFD-transplanted with fecal microbiota from lean rats (HFGFD-FMT, *n* = 11). HFGFD-VEH individuals received oral probing vehicle (sterile PBS), while individuals in HFGFD-CON and HFGFD-FMT groups were subjected to different microbiota-based treatments, which consisted of oral administration of a bacterial consortium of defined composition and fecal microbiota transplantation (FMT), respectively (see further). The consortium treatment started after 8 weeks of HFGFD intervention, and it consisted of the administration of the following nine human gut commensal strains: *Faecalibacterium prausnitzii*, *Butyricicocccus pullicaecorum*, *Roseburia inulinivorans*, *Akkermansia muciniphila*, *Lactiplantibacillus plantarum* (former *Lactobacillus plantarum*), *Anaerostipes caccae*, *Phocaeicola vulgatus* (former *Bacteroides vulgatus*), *Veillonella parvula*, and *Blautia obeum*. On each dosing day, lyophilized consortium was washed and reconstituted in anaerobic DPBS (5340 g, 10 min, ambient), in an anaerobic hood (N2:CO2:H2; 80:10:10). As a result, a concentrated liquid, ready-to-use formulation in air-tight vials, was obtained. This suspension was administered immediately. Animals were dosed daily with 2 × 10^9^ CFU (total counts of the 9 strains), using a disposable oral probe (Biochrom Ltd., Cambridge, UK); this procedure was repeated every 24 h for 2 weeks. For individuals belonging to the vehicle group, the same procedure was followed, but sterile DPBS was administered instead. Rats receiving the bacterial consortium were housed individually throughout the treatment period to avoid cross-contamination.

Fecal microbiota transplantation (FMT) was also performed after 8 weeks of HFGFD intervention. To achieve higher homogeneity in the transplantation procedure, feces from three control (lean) rat donors were pooled to be used for transplantation. With the aim of reducing gastric acidity and thus increasing the survival of the microorganisms, omeprazole was administered orally at a dose of 50 kg/day during the 3 days prior to intestinal decontamination. To proceed with intestinal emptying, rats were maintained in isolation in fast grills, and two oral doses of CitraFleet (sodium picosulfate, 0.16 mg/mL and magnesium oxide 51.2 mg/mL) of 1 mL and 2 mL were administered 24 h and 12 h, respectively, before the transplant. These administrations were accompanied by 2 mL of water each. Recolonization was performed by a single oral gavage, for which 100 mg of the fecal pool were dissolved in 2 mL of sterile DPBS. In both the consortium and FMT groups, the treatment period lasted 2 weeks during which animals were maintained on the original diet (Figure 1A). Rats were housed under a 12 h light/dark cycle at constant temperature (24 ± 1 °C) and relative humidity (55 ± 10%). At week 10, liver hemodynamics and blood biochemistry were performed, and liver tissue samples were collected for histological and molecular analyses. In addition, the cecum content was collected for shotgun sequencing of gut microbes (Supplementary Materials). For metagenomics analysis, fecal samples were also obtained from a group of rats receiving a regular diet to compare HFGFD-induced changes on microbial composition with those of rats fed a control diet (CD).

### 2.2. STAM™ Mouse Model of NASH

The STAM™ study was conducted by SMC Laboratories in Japan, following previously reported protocols [12,13]. Briefly, NASH was induced in C57 BL/6J male mice by a single subcutaneous injection of 200 μg streptozotocin (STZ, Sigma-Aldrich, St. Louis, MO, USA) 2 days after birth, to induce mild pancreatic islet inflammation and destruction (Figure 1B). At 4 weeks of age, the animals initiated a high-fat diet (HFD, 57 kcal% fat, Cat# HFD32, CLEA Japan, Inc., Tokyo, Japan). Animals were randomized into three treatment groups based on their body weight the day before initiating the HFD. At 5 weeks of age (after one week on HFD), the animals received either sham gavage (STAM + VEH, *n* = 16), the nine-strain bacterial consortium (STAM + CON, *n* = 18), or Telmisartan (STAM + TLM, *n* = 8), an angiotensin II receptor blocker that decreases hepatic fat accumulation and inhibits hepatic stellate cell activation and thus suppresses hepatic fibrogenesis [14]. Vehicle (sterile PBS) and bacterial consortium were administered orally in a volume of 200 µL/mouse; the consortium was administered at the dose of 10^9^ CFU/day (total counts of the 9 strains) for a total of 4 weeks. Product suspensions were prepared as described above. Telmisartan was used as benchmark and administered orally at the daily dose of 10 mg/kg in a volume of 10 mL/kg for 4 weeks. For that, one tablet of Telmisartan was transferred into a mortar and triturated with pestle by adding pure water until obtaining a homogenous suspension of 1 mg/mL. All treatments were prepared freshly prior to administration. A control group fed a regular diet and receiving sham gavage was also included in this study (CD + VEH, *n* = 8) (Figure 1B). Body weight was recorded daily during the experimental period. Animals were maintained in a SPF facility under controlled conditions of temperature (23 ± 3 °C), humidity (50 ± 20%), lighting (12 h artificial light and dark cycles; light from 8:00 to 20:00), and air exchange.

Animals belonging to the control (CD + VEH, *n* = 8) and STAM groups (STAM + VEH, STAM + CON, and STAM + TLM, *n* = 8, *n* = 10, or *n* = 8, respectively) were sacrificed after 4 weeks of treatment, i.e., at 9 weeks of age, which corresponds to a steatohepatitis/early fibrosis stage. In addition, two groups of STAM animals belonging to the vehicle (*n* = 8) and consortium (*n* = 8) groups were followed for three additional weeks and were sacrificed at 12 weeks of age, which corresponds to a stage of late fibrosis. This was performed to investigate the potential of the bacterial consortium to delay disease progression upon cessation of treatment (Figure 1B). Animals were sacrificed by exsanguination through direct cardiac puncture under isoflurane anesthesia (Pfizer Inc., Tokyo, Japan). Blood was collected for biochemistry, and the whole liver for immunohistochemistry and histopathology.

### 2.3. Blood Biochemistry

Blood samples from fasting rats were collected from the cava vein at sacrifice. Glucose, bilirubin, alanine aminotransferase (ALT), aspartate aminotransferase (AST), cholesterol, high-density lipoprotein (HDL), low-density lipoprotein (LDL), triglycerides, and albumin were measured with standard methods at the Hospital Vall d’Hebron CORE lab. Insulin was measured using an ELISA kit following the manufacturer’s instructions (EMD Millipore, Billerica, MA, USA). Insulin resistance was estimated by applying the homeostasis model of insulin resistance index (HOMA-IR): (fasting insulin (ng/mL) × fasting glucose (mg/dL))/405.

In the STAM™ mice, non-fasting blood was drawn from the facial vein for the quantification of glycated hemoglobin (HbA1c), cytokeratin (CK)-18, and biochemistry. HbA1c levels were quantified in whole blood by DCN2000 + (Siemens Healthcare Diagnostics, USA). Serum CK-18 levels were measured by using the Mouse Cytokeratin 18-M30 ELISA kit (Cusabio Biotech Co., Ltd., Wuhan, China). Serum alanine aminotransferase (ALT) and triglyceride levels were measured by FUJI DRI-CHEM 7000 (Fujifilm Corporation, Tokyo, Japan). Serum total cholesterol, high-density lipoprotein (HDL)-cholesterol, and low-density lipoprotein (LDL)-cholesterol levels were quantified by HPLC at Skylight Biotech Inc. (Akita, Japan).

### 2.4. Histological Analyses

Animal liver samples were extracted, fixed in 4% formalin, embedded in liquid paraffin at 65 °C, and sectioned in 4 μm thick slices (rats) or fixed in Bouin´s solution (Sigma-Aldrich Japan, Tokyo, Japan) (mice). Samples were then stained with hematoxylin and eosin (H&E) to assess liver parenchyma, or with Sirius red to detect collagen fibers. All stained liver samples were examined by an expert liver pathologist blinded to the interventions.

The diagnosis of NASH in rats was established based on the presence of all three characteristic patterns of the disease, which include the coexistence of steatosis, lobular inflammation, and hepatocellular ballooning. The NASH-CRN Activity Score (NAS) was used to quantify NASH activity. NAS comprises the unweighted sum of the histological components: steatosis (0–3), lobular inflammation (0–3), and hepatocellular ballooning (0–2). Fibrosis was also classified in five stages according to the NASH-CRN system, ranging from F0 (no fibrosis) to F4 (cirrhosis). NAFLD Activity score (NAS) of STAM™ mice was calculated according to the criteria of Kleiner [15].

### 2.5. Liver Hemodynamics

The hemodynamic measurements were performed in fasted rats under intraperitoneal anesthesia with ketamine (100 mg/kg) plus midazolam (5 mg/kg) and body temperature maintained at 37 °C. Mean arterial pressure (MAP) was measured by catheterization of the femoral artery and portal pressure (PP) assessed by ileocolic vein catheterization using highly sensitive pressure transducers (Harvard apparatus, Holliston, MA, USA). Superior mesenteric artery (SMA) blood flow (SMABF, mL/(min × 100 g)) and portal blood flow (PBF, mL/(min × 100 g)) were measured with a perivascular ultrasonic transit-time flow probe (1 mm diameter, Transonic systems Inc, Ithaca, NY, USA). SMA resistance (SMAR) and intrahepatic vascular resistance (IHVR, mmHg/mL × min × 100 g) were calculated as ((MAP-PP)/SMABF) and (PP/PBF), respectively.

### 2.6. Western Blot

Rat livers were perfused with saline for exsanguination and samples were directly frozen in liquid nitrogen and stored at −80 °C until further use. Then, liver samples were crushed cold and homogenized in Triton-lysis buffer, sonicated, and centrifuged at 14,000 rpm for 10 min at 4 °C. Supernatant total protein concentration was quantified by a BCA protein assay kit (ThermoFisher Scientific, Waltham, MA, USA). Forty (40) micrograms of protein was run on a 10% sodium dodecyl sulphate–polyacrylamide gel electrophoresis (SDS-PAGE). Proteins separated by SDS-PAGE were transferred onto a polyvinylidene fluoride (PVDF) membrane (ThermoFisher Scientific, Waltham, MA, USA). Membranes were washed with TTBS 1X several times and blocked for 1 h at RT with PhosphoBLOCKERTM 5% (Cell biolabs, San Diego, CA, USA) to detect the phosphorylated proteins P-Akt (1/500, Cell signaling, Danvers, MA, USA) and P-eNOS (Ser1177, 1/250), or with skimmed milk to detect Kruppel-Like Factor 2 (Klf2) (1/200, Santa Cruz biotechnology, Dallas, TX, USA). Gapdh antibody (1/5000, Ambion, Austin, TX, USA) was used as the loading control. Membranes were developed using the ECL kit (GE Healthcare; Little Chalfont, UK) and protein expression was finally determined by densitometry analysis bands using Image Studio Lite (Lincoln, NE, USA).

### 2.7. Real-Time PCR

Rat liver samples were maintained at least 24 h in RNA*later* (ThermoFisher Scientific, Waltham, MA, USA) and then stored at −80 °C until further use. Total RNA was extracted by using the RNeasy mini-Kit (QIAGEN, Venlo, The Netherlands), and retro-transcribed to complementary DNA (High-capacity cDNA reverse transcription, ThermoFisher Scientific, Waltham, MA, USA). Twenty (20) nanograms of cDNA was added to Taqman universal PCR master mix plus the specific Taqman probes for *α-Sma* (alpha-Smooth muscle actin, Reference code: Rn01759928_g1) and *Col1a1* (Collagen type I alpha 1 chain, Reference code: Rn01463848_m1) (Life Technologies Ltd., Renfrew, UK). Quantitative reverse-transcription polymerase chain reaction (qRT-PCR) was performed using the 7900HT Fast Real-Time PCR system (ThermoFisher Scientific, Waltham, MA, USA), based on a standard protocol of 40 cycles at 95–60 °C. The relative gene expression was normalized to *β-Actin*. Data were analyzed using the Relative Quantification qPCR Application in ThermoFisher Cloud. For results calculation, the 2^–∆∆Ct^ method was used.

### 2.8. Biological Parameter Analyses

Continuous variables were tested for normality using the D’Agostino–Pearson normality test and are documented as mean ± standard error of the mean (SEM). Statistical analyses were performed using GraphPad Prism software (GraphPad Software, San Diego, CA, USA). Biological parameters of treatment groups were compared by Student’s *t*-test or One-Way ANOVA with Dunnett’s multiple comparisons’ test. A *p*-value < 0.05 was considered statistically significant.

## 3. Results

### 3.1. The Nine-Strain Bacterial Consortium Improves Portal Hypertension (PHT), Endothelial Dysfunction (ED), and Fibrotic Markers in the Rat NASH Model of PHT

At the end of the 2-week treatment period, body weight (BW), blood biochemistry, NASH histology, and systemic and portal hemodynamics were evaluated to elucidate the effects of the bacterial consortium treatment and FMT on the regulation of PHT and ED in NASH rats.

#### 3.1.1. Microbiota-Based Treatments Significantly Improved Body Weight

The HFGFD intervention induced a marked increase in BW (Table 1 and Figure S1A). During the 2-week treatment period, animals in the HFGFD-VEH group continued increasing their BW to a great extent (22.08 ± 3.18 g). However, HFGFD-CON only gained on average 2.91 ± 3.55 g (*p* < 0.05), whereas HFGFD-FMT lost on average 5 ± 3.47 g (*p* < 0.05). Compared to the HFGFD-VEH, both groups receiving microbiota-based treatments gained significantly less weight during the 2-week treatment.

#### 3.1.2. The Metabolic Profile Improved in the Treatment Groups

Both microbiota-based treatments displayed an important reduction in fasting blood glucose and insulin levels, and HOMA-IR index with respect to the HFGFD-VEH group, although these differences did not reach statistical significance (Table 1 and Figure S1B,C). Both treatments showed a significant but slight increase in triglycerides when compared to the vehicle group. Cholesterol fractions remained unchanged (Table 1). ALT levels were significantly reduced, and albumin was significantly improved in the FMT group.

#### 3.1.3. No Treatment Group Significantly Reversed the NASH Histological Pattern

**Figure 2:**
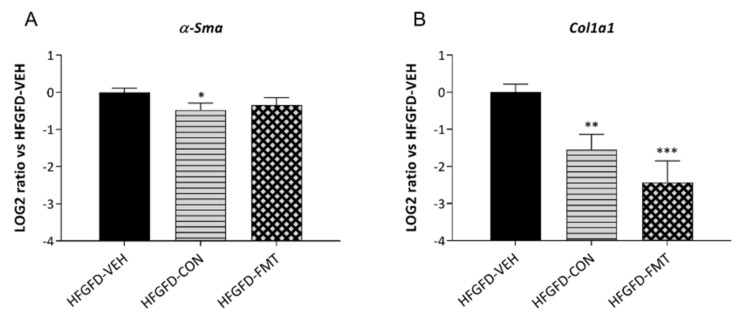
Relative mRNA expression of (**A**) *α-Sma* and (**B**) *Col1a1* measured by quantitative RT-PCR in the liver of NASH rats and expressed as log2 ratio. *β-Actin* was used as an endogenous control, and results were normalized to the HFGFD-VEH group. HFGFD-VEH: group of NASH rats receiving sham gavage (vehicle); HFGFD-CON: group of NASH rats receiving the 9-strain bacterial consortium daily; HFGFD-FMT: group of NASH rats receiving fecal microbiota transplantation from control lean rats (1× transplantation followed by sham gavage). Abbreviations: α-Sma, alpha-Smooth muscle actin; Col1a1, Collagen type I alpha 1 chain. *, **, *** *p* ≤ 0.05, ≤0.01, and ≤0.001, respectively, versus HFGFD-VEH.

HFGFD intervention for 10 weeks resulted in a consistent reproduction of NASH histological hallmarks (i.e., co-occurrence of steatosis, hepatocellular ballooning, and lobular inflammation) (Figures S2 and S3). However, none of the 2-week microbiota-based treatments were associated with significant improvements in overall NASH histological diagnosis or its individual features. This is in line with previously obtained results following microbiota transplantation in this particular NASH model [11]. Interestingly, no animals receiving HFGFD-CON presented inflammation or ballooning scores above 1 (Figure S2B,C). Finally, as shown in the Sirius red-stained liver sections in Figure S3, none of the groups developed fibrosis. This is also in agreement with previous results [11]. However, expression of the pro-fibrotic hepatic marker *Col1a1* was significantly reduced in the microbiota-based treatments when compared with the vehicle group; in contrast, *α-SMA* was only reduced by the consortium treatment (Figure 2).

#### 3.1.4. Microbiota-Based Treatments Significantly Reduced Portal Pressure and Improved Liver Hemodynamics

Consistent with previous findings [11,16], and despite the absence of histological fibrosis, animals in the 10-week HFGFD intervention showed values of portal pressure (PP) of 10.32 mmHg on average (Table 2). Importantly, both the bacterial consortium treatment and FMT significantly reduced PP when compared to the vehicle-treated group, even while maintaining the HFGFD during the 2-week treatment period (Table 2). This decrease in PP observed in the HFGFD-CON group was secondary to a significant decrease (80% reduction) in intrahepatic vascular resistance (IHVR) as compared to HFGFD-VEH, in a similar degree as measured in the HFGFD-FMT group (104% reduction) (Table 2). Regarding systemic hemodynamics, no relevant differences were observed between groups (Table 2).

#### 3.1.5. Features of ED Were Improved in Animals after Receiving Microbiota-Based Treatments

**Figure 3:**
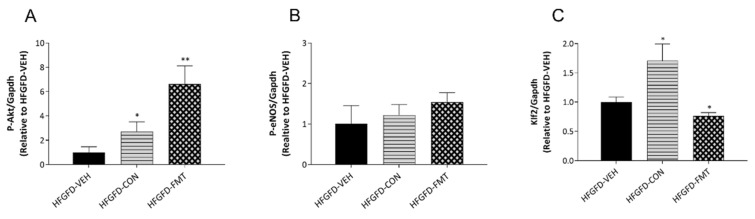
Expression of markers of endothelial dysfunction in NASH rats. (**A**–**C**) Western blot analysis of intrahepatic markers of endothelial dysfunction. Bar diagrams show the quantification of phosphorylated (P)-Akt, P-eNOS, and Klf2 using glyceraldehyde 3-phosphate dehydrogenase (Gapdh) as a loading control and normalized to the HFGFD-VEH group. HFGFD-VEH: group of NASH rats receiving sham gavage (vehicle); HFGFD-CON: group of NASH rats receiving the 9-strain bacterial consortium daily; HFGFD-FMT: group of NASH rats receiving fecal microbiota transplantation from control lean rats (1× transplantation followed by sham gavage). Abbreviations: Akt, Protein kinase B; eNOS, Endothelial nitric oxide synthase; Klf2, Kruppel-Like Factor 2. *, ** *p* ≤ 0.05 and ≤0.01, respectively, versus HFGFD-VEH.

ED molecular hallmarks were assessed to further characterize the changes observed in IHVR and PP induced by the microbiota-based treatments with respect to the vehicle-treated group [17,18]. As seen in Figure 3, microbiota-based treatments induced a significant increase in P-Akt. P-eNOS showed a tendency to increase in both HFGFD-CON and HFGFD-FMT when compared to HFGFD-VEH, although it did not reach statistical significance. Interestingly, only the consortium-treated group showed a marked significant increase in Klf2 (Figure 3C).

#### 3.1.6. Microbiota-Based Treatments Improved Cecal Species Diversity and Induced Composition Shifts

**Figure 4:**
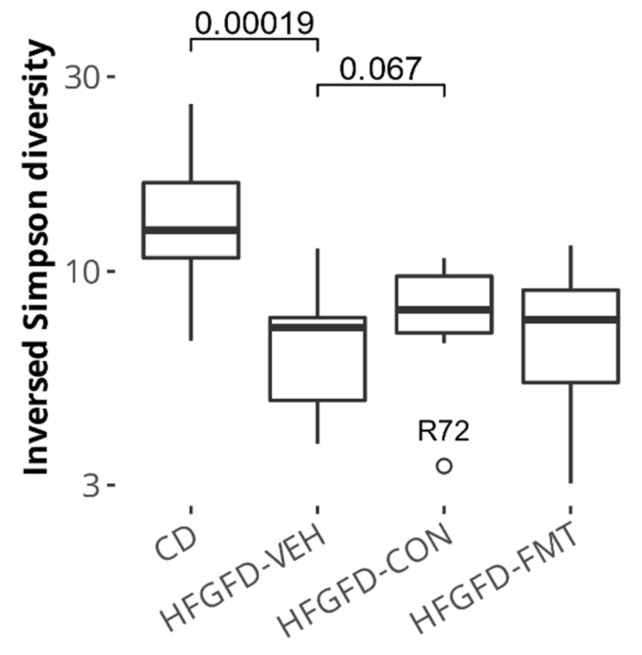
Microbial diversity in the cecum of NASH rats. Statistically significant differences are depicted above boxplots; these represent Wilcoxon signed–rank test ***p***-values computed without the outlier R72. HFGFD-VEH: group of NASH rats receiving sham gavage (vehicle); HFGFD-CON: group of NASH rats receiving the 9-strain bacterial consortium daily; HFGFD-FMT: group of NASH rats receiving fecal microbiota transplantation from control lean rats (1× transplantation followed by sham gavage).

To verify the effect of both microbiota-based treatments on (bacterial) species diversity and taxonomic composition, the cecum content of rats was collected at sacrifice, and microbial DNA was extracted and sequenced. The Inverse Simpson diversity index revealed that, although not reaching the microbial diversity levels of the control diet (CD) group, diversity improved over vehicle (HFGFD-VEH) in both treatment groups; this was nearly statistically significant in the bacterial consortium (HFGFD-CON) treatment group (Figure 4).

Classifier performance as analyzed through balanced error rate (BER) suggested that HFGFD-CON induced a more defined change than HFGFD-FMT (Figure S4). In addition, the corresponding feature selection (VIP > 1, *n* = 123) showed that the bacterial consortium treatment induced the suppression of 72 (58.5% of observable change) vehicle-associated taxa and promoted the emergence of 48 (41.5%) non-product treatment associated taxa, whereas FMT, according to feature selection (*n* = 0), induced the suppression of 135 (75.8%) taxa, and replacement with 43 (24.2%) taxa. Hence, both treatments produced an important replacement of the HFGFD-VEH associated microbial community, but the bacterial consortium (CON) achieved a more defined and richer replacement than FMT. Figure S4B provides the top 10 discriminant taxa according to classifier estimated variable importance (VIP) and their associations, whereas Figure S4C combines the three VEH/CON, VEH/FMT and CON/FMT contrasts into a single Euler plot proportional to the numbers of features selected for each contrast.

#### 3.1.7. The Nine-Strain Bacterial Consortium Induced Functional Microbiome Shifts

**Figure 5:**
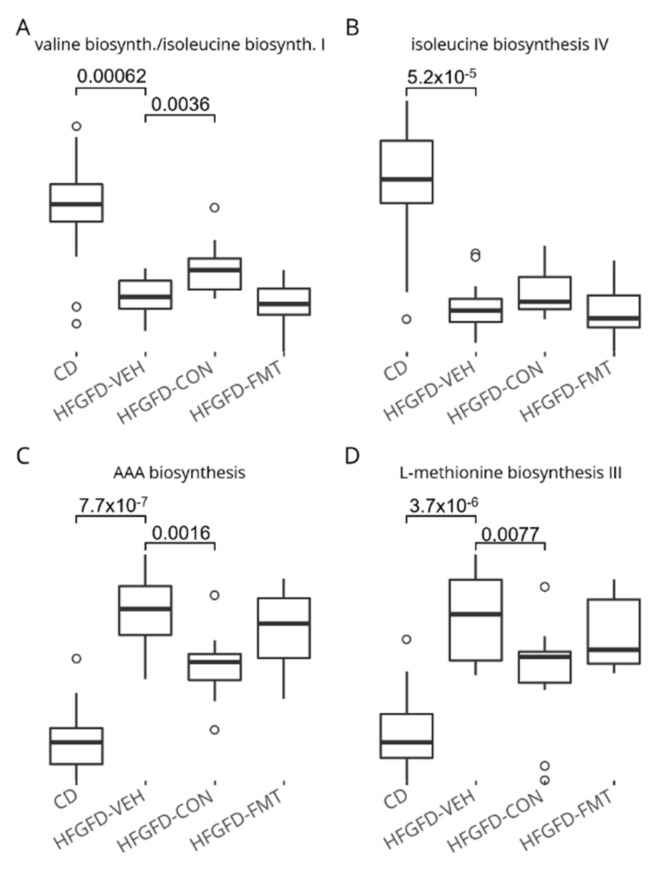
Predicted BCAA (**A**,**B**), AAA (**C**), and Methionine (**D**) biosynthesis pathways in the cecum of NASH rats. Statistically significant differences are depicted above boxplots. HFGFD-VEH: group of NASH rats receiving sham gavage (vehicle); HFGFD-CON: group of NASH rats receiving the 9-strain bacterial consortium daily; HFGFD-FMT: group of NASH rats receiving fecal microbiota transplantation from control lean rats (1× transplantation followed by sham gavage).

Changes in microbial composition, as evaluated by whole metagenome shotgun (WMS) sequencing, were used to conduct predictive pathway analysis to unravel putative biosynthetic pathways that could be modulated by treatment. The bacterial consortium (CON) treatment was predicted to drive an increase in microbial branched-chain amino acids (BCAA) and a decrease in aromatic amino acids (AAA) biosynthesis capacities. In addition, methionine biosynthesis was also predicted to decrease. These changes over HFGFD-VEH were significant in the HFGFD-CON but not in the HFGFD-FMT group (Figure 5).

#### 3.1.8. Microbiota-Based Treatments Induced Differential Hepatic Gene Expression

The expression of 20,359 liver genes was documented through RNA-Seq analysis of the three HFGFD groups. Of these, 444 (2.2%) were considered discriminant by sPLS-DA analysis for one or more of the CON/VEH, FMT/VEH or CON/FMT contrasts, with most genes being induced by treatment. Figure S5C depicts the breakdown on up- vs. downregulated discriminant genes. Table 3 provides details on gene expression modulation by the microbiota consortium of the top 1000 fatty liver associated genes according to the Harmonizome database, and eventual FMT equivalent. We have detected molecular chaperones to be predominantly modulated by both microbiota-based treatments. Among all modulated chaperones, we highlight the heat shock protein (Hsp) family members *Hspa1a*, *Hspa1b*, and *Hspa8*, as these were strongly downregulated by both treatments. Microbiota treatments also induced the downregulation of the endoplasmic reticulum (ER) chaperone BiP (*Hspa5*), as well as of its co-chaperone *Dnajb9*. Additional key genes downregulated by treatment were the cell division cycle *(Cdc)42* and the Rac family small GTPase *(Rac)1* (Table 3). The RNA-seq analysis also revealed that both microbial treatments enhanced the expression of several putative tumor suppressor genes (data not shown).

Among the upregulated genes, the thyroid hormone responsive Spot14 (*Thrsp*) gene was found to be one of the main modulated genes, as both treatments considerably increased its expression. In addition, the AKT Serine/Threonine Kinase 1 (*Akt1*) was found to be only upregulated by the microbiota consortium (CON) treatment (Table 3).

Finally, over-representation analysis of differentially expressed genes induced by the microbiota consortium treatment was performed. Up- and downregulated genes were considered separately and both a restrictive (VIP > 1, pFDR < 0.1) and a relaxed (pFDR < 0.1) condition was used as input, generating respectively, 281 up- and 191 downregulated genes, and 489 up- and 326 downregulated genes. Consistent with the observed amelioration of the hepatic pro-fibrotic marker *Col1a1* (Figure 2B), liver transcriptomics identified numerous pro-fibrotic pathways being positively modulated by the two microbiome-based treatments. In this regard, we highlight the pro-fibrotic signaling pathways VEGFA-VEGFR2 (WP3888), Wnt signaling (WP428), and the Cytoskeletal regulation by Rho GTPases (P00016), all found to be putatively downregulated by the microbiota consortium (CON) treatment (Table 4).

### 3.2. The Nine-Strain Bacterial Consortium Delays Disease Progression and Improves NAFLD Disease Markers in the STAM™ Mouse Model

Because liver transcriptomics performed in the NASH rats suggested that the tested bacterial consortium could be favorably modulating pro-fibrogenic pathways (Table 4), we were prompted to test the same consortium on a rodent model that displays notable signs of fibrosis at histopathology. For that, the STAM™ mouse model was used. The therapeutic effect of the nine-strain consortium was evaluated and compared to the benchmark Telmisartan. The effects were evaluated at the histopathological level and blood was collected for biochemical analysis. Because STAM animals are lean [19], as expected, the bacterial consortium did not improve BW (data not shown). As observed in the NASH rats, no significant alterations were detected in blood parameters, namely ALT and cholesterol levels (Table 5). However, a trend of decreased whole blood levels of glycated hemoglobin (HbA1c) was observed by the end of the study (Figure S6).

#### 3.2.1. The Consortium of Nine Gut Commensals Improved NAS at Histopathology at 9 Weeks of Age

**Figure 6:**
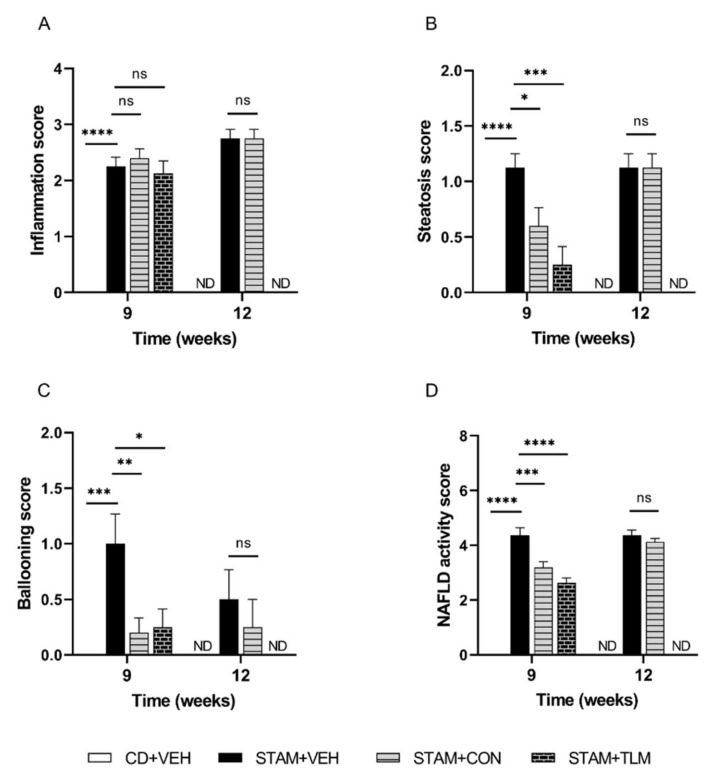
NAFLD activity score (NAS) in the STAM™ study. Histopathology was evaluated in H&E-stained liver sections, and the NAS score was calculated. The components (**A**) inflammation, (**B**) steatosis, (**C**) ballooning, and (**D**) the composite score, were evaluated at both 9 and 12 weeks of age (euthanasia 1 and 2, respectively). CD + VEH: group of control diet mice receiving sham gavage (vehicle); STAM + VEH: group of STAM mice receiving sham gavage (vehicle); STAM + CON: group of STAM mice receiving the 9-strain bacterial consortium daily; STAM + TLM: group of STAM mice receiving Telmisartan daily. ND: not determined for the CD + VEH and STAM + TLM groups at 12 weeks. ns: not significant. *, **, ***, **** *p* ≤ 0.05, ≤0.01, ≤0.001, and ≤0.0001, respectively, versus STAM + VEH.

As expected [13], liver sections from the vehicle-treated STAM group exhibited micro- and macrovesicular fat deposition, hepatocellular ballooning, and inflammatory cell infiltration (Figure 6 and Figure S7). The bacterial consortium-treated STAM mice (STAM-CON) displayed significantly reduced NAS scores when compared to the disease group at 9 weeks of age (Figure 6D). This was the result of reduced steatosis and ballooning scores (Figure 6B,C). At 12 weeks of age this was no longer observed.

#### 3.2.2. The Consortium of Nine Gut Commensals Improved Fibrosis, and Showed Reduced Hepatic Expression of F4/80 and Serum CK-18 Levels

**Figure 7:**
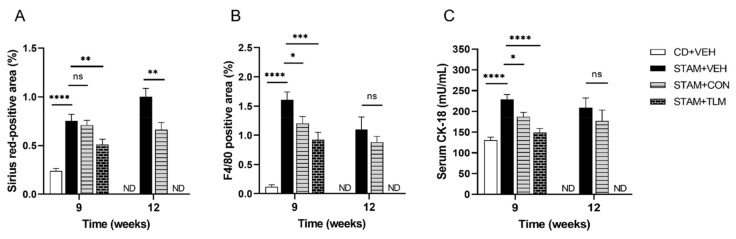
Hepatic fibrosis area, F4/80 positive area, and serum CK-18 levels in the STAM™ study. (**A**) Fibrosis assessed in Sirius red-stained liver sections. (**B**) Immunohistochemistry of macrophages evaluated in F4/80-immunostained sections. (**C**) Serum levels of CK-18, a marker for apoptotic hepatocytes. All markers were evaluated at both 9 and 12 weeks of age (euthanasia 1 and 2, respectively). CD + VEH: group of control diet mice receiving sham gavage (vehicle); STAM + VEH: group of STAM mice receiving sham gavage (vehicle); STAM + CON: group of STAM mice receiving the 9-strain bacterial consortium daily; STAM + TLM: group of STAM mice receiving Telmisartan daily. ND: not determined for the CD + VEH and STAM + TLM groups at 12 weeks. ns: not significant. *, **, ***, **** *p* ≤ 0.05, ≤0.01, ≤0.001, and ≤0.0001, respectively, versus STAM + VEH.

To evaluate collagen deposition, liver sections were stained with Sirius red (Figure 7A and Figure S8A). As expected, liver sections from the vehicle-treated STAM group (STAM-VEH) displayed increased collagen deposition in the pericentral region of the liver lobule when compared to the control diet group. The bacterial consortium-treated group (STAM-CON) showed a significant decrease in the fibrosis (Sirius red-positive) area when compared to the disease group at 12 weeks of age but not at 9 weeks of age, when the amount of fibrosis is lesser. This therapeutic effect on fibrosis was also confirmed on fibronectin immunostained liver sections (Figure S9).

To further evaluate inflammation, macrophage F4/80 immunostaining was used in liver sections. Even though no significantly reduced general inflammation was observed in H&E-stained sections (Figure 6A and Figure S7), the F4/80-stained sections showed a significant reduction in macrophage inflammatory infiltration in the consortium-treated group at 9 weeks of age, and a trend to decrease at 12 weeks of age (Figure 7B and Figure S8B). Finally, CK-18 serum levels were also significantly reduced in the consortium-treated group at 9 weeks of age, and a trend to decrease was observed by 12 weeks of age (Figure 7C), a result that correlates with reduced hepatocyte apoptosis and improved histological activity [20].

## 4. Discussions

The gut–liver axis has a recognized role in NASH onset and progression, possibly fueled by the anatomo-functional crosstalk between the intestine and the liver. It is well described that a disrupted intestinal barrier integrity is frequently observed in NAFLD patients, and that the ensuing state of low-grade inflammation and endotoxemia, due to the translocation of harmful gut-born molecules, correlates with disease severity [21]. However, the effects of bacterial-derived molecules go beyond the translocation of microbe-associated molecular patterns (MAMPs) such as lipopolysaccharides (LPS), and numerous metabolites resulting from bacterial fermentation have been described to contribute to pathology [22]. Therefore, therapeutic approaches aimed to restore and balance the gut microbiome are considered a promising strategy to treat NASH and other liver diseases [23,24]. In this regard, FMT has been shown to improve disease pre-clinically in NASH rats, by restoring the insulin/p-Akt/p-eNOS signaling pathway, thereby improving IR and ED [11]. In humans, this treatment modality is also being explored [24,25], and although so far, no severe adverse events have been reported in NAFLD-FMT trials, several factors may hamper its clinical applicability such as the amount and frequency of stool transplantation, the need for bowel preparation prior to treatment, heterogeneity of fecal donors, and long-term expected effects [22]. Therefore, consortia of defined bacterial composition are a valuable alternative [22,23,24].

Relevant research, conducted in different animal models, has been previously performed using combinations of defined bacterial strains as therapeutic strategies in the management of NASH [23,26,27,28]. In the current study, we have tested a nine-strain consortium of gut commensals of defined composition and amount. Among others, the tested consortium was composed of four butyrate producers (*Faecalibacterium prausnitzii*, *Butyricicocccus pullicaecorum*, *Roseburia inulinivorans*, and *Anaerostipes caccae*) and five propionate producers (*Roseburia inulinivorans*, *Akkermansia muciniphila*, *Phocaeicola vulgatus*, *Veillonella parvula*, and *Blautia obeum*). We tested this consortium in a therapeutic setting in two rodent models of NASH: the rat NASH model of PHT, and the STAM™ mouse model. In rats, the consortium showed a protective effect on BW, a trend to improve fasting blood insulin levels and HOMA-IR, and a significant protective effect on PHT, namely a significant reduction in IHVR and PP. Similar to previous results [11] and to the FMT group included in the current study, protection occurred through the restoration of the sensitivity to insulin of the hepatic Akt-dependent eNOS signaling pathway. In addition, an upregulation of Klf2 was observed in the liver of consortium-treated rats. This is also relevant, as the upregulation of hepatic Klf2 has been shown to ameliorate PHT in cirrhotic rats, via inactivation and apoptosis of hepatic stellate cells, together with a reduction in oxidative stress and improvement in endothelial function [17,18]. Despite these results, we did not observe improvement in NASH at histopathology. Nevertheless, this is also in accordance to previous studies testing the efficacy of stool transplantation in this model, specifically developed to evaluate diet-induced endothelial dysfunction and PHT in NASH, rather than NASH histopathology [11].

Previously, FMT has been shown to significantly alter the gut microbial composition of NASH rats [11]. However, FMT is preceded by intestinal emptying before stool transplant, which, in the current study, resulted in the suppression of numerous microbial taxa as opposed to the consortium treatment. Thus, we observed that the bacterial consortium induced more defined and richer changes in the gut microbiota than FMT. When translating these taxonomic shifts into predictive metabolic alterations, namely putative changes on microbial metabolic pathways, results suggested that the consortium was predicted to increase the biosynthesis of BCAA, and to decrease the biosynthesis of AAA and methionine. This is an interesting result, because a decreased BCAA/AAA ratio has been shown to correlate with liver dysfunction in cirrhotic patients [29]. Whether an altered fecal amino acid profile contributes to NAFLD disease progression in the current study remains to be determined. Nonetheless, BCAA treatment was shown to ameliorate liver fat accumulation in experimental animal models via increased production of acetic acid by gut microbes [30]. Therefore, this warrants further investigation, to determine whether the nine-strain bacterial consortium can effectively increase the levels of BCAA and decrease those of AAA as predicted, and thereby ameliorate disease.

The administration of the nine-strain consortium also altered the hepatic gene expression. Several disease-associated genes were downregulated by this treatment. Among these, several members of the heat shock family of proteins (HSP) were strongly downregulated by both the consortium and FMT treatments, including *Hspa1a*, *Hspa1b*, *Hspa8*, and *Hspa5*, all members of the Hsp70 family of proteins, and the Hsp40 co-chaperone *Dnajb9*. HSP are produced in response to stress and play an important role in assisting protein synthesis from the secretory pathways of the endoplasmic reticulum (ER), to ensure correct protein folding [31]. ER stress is involved in the progression of NAFLD to NASH and abnormal ER stress responses have direct pathological consequences, including fat accumulation, IR, inflammation, apoptosis, and, consequently, fibrosis [32]. Of importance, drugs targeting HSP as anti-fibrotics in NASH are currently in Phase II of clinical development (ClinicalTrials.gov NCT04267393). It is therefore conceivable that the observed downregulation of HSP by the microbiota-based treatments in our study reflects a diminished ER stress response. Among the upregulated genes, *Thrsp* (encoding Spot14) was found to be induced by both treatments. Spot14 (S14) was originally identified as a mRNA from rat liver that responded rapidly to thyroid hormone [33]. Recently, it has been described to play a key role in the tissue-specific regulation of lipid metabolism in response not only to thyroid hormone but also to dietary substrates such as glucose and polyunsaturated fatty acids, and also to other hormones such as insulin and glucagon [34]. Spot14 is involved in de novo synthesis of fatty acids, and in the export of lipids from the liver as very low-density lipoprotein (VLDL) particles [34]. Why the currently tested microbiota-based treatments would induce S14 transcription is not clear, but it may reflect the increased hepatic responsiveness to insulin, as suggested by studies performed both in rodents and obese patients [35,36,37].

Even though histological changes in fibrosis cannot be addressed in the NASH-PHT rat model, some pro-fibrotic markers such as *α-Sma* and *Col1a1* were found to be downregulated upon treatment with the nine-strain consortium. In addition, liver transcriptomics analysis suggested that a few fibrogenic pathways were downregulated by the treatment, such as the VEGFA-VEGFR2, the Wnt signaling, and the Cytoskeletal regulation by Rho GTPases pathways [38]. However, because histological fibrosis cannot be adequately tested in the NASH-PHT rat model, we tested the same nine-strain consortium in a rodent model which developed liver fibrosis: the STAM™ mouse model. In this study, the consortium-treated animals showed anti-fibrotic effects, and significantly reduced collagen- and fibronectin-positive areas in liver sections at 12 weeks. Although a trend to decrease at 9 weeks was also observed, this did not reach statistical significance, possibly because of the lower fibrosis levels developed at that time point. In addition, consortium-treated animals also displayed a reduced F4/80 positive area, a marker for mouse macrophages, as well as lower levels of serum CK-18, a marker of hepatocyte apoptosis, thereby suggesting an overall beneficial effect on all key aspects of NASH pathology. Of further relevance is that the consortium dosage stopped at 9 weeks of age. This may explain, in part, the lesser protective effects observed at 12 weeks in histological steatosis, ballooning, and inflammation, especially when compared with the clear beneficial effects at 9 weeks, thus suggesting a reversal of these more dynamic readouts upon treatment withdrawal. However, it also showed that the protective effect observed at the level of fibrosis at 12 weeks of age was long-lasting, and still apparent 3 weeks after the cessation of treatment. It is still to be evaluated whether these therapeutic effects would be even more pronounced if the consortium had been administered until sacrifice at 12 weeks.

The currently tested consortium is composed, among others, of several butyrate and propionate producers. A non-significant increase in cecal butyrate and propionate levels was measured in the NASH rats (data not shown). Whether these account for the observed benefits in this study is not known. However, sodium butyrate supplementation has been shown to attenuate high-fat diet-induced NASH in mice, most likely by restoring gut dysbiosis and improving gastrointestinal barrier [39]. Interestingly, overweight and obese patients also have higher levels of propionate in their stools, suggesting that either propionate is overproduced, or it is mal-absorbed [40]. In addition, in overweight adult humans, propionate has been shown to prevent weight gain and insulin resistance [41]. Although the role of SCFA in liver health is not completely understood, it has been suggested that when the balance between caloric intake and expenditure is maintained, SCFAs would benefit liver health [42]. Therefore, it is reasonable to postulate that this consortium may improve disease partially due to increased butyrate and propionate levels in the intestine.

Further work must be performed to recapitulate the findings in the two models, namely in what concerns microbial changes in the gut lumen, as well as liver molecular alterations, to confirm the mode-of-action behind the consortium treatment. Nonetheless, the current results support the use of defined combinations of gut commensal bacteria for the treatment of NASH, so to delay disease progression.

## 5. Conclusions

Despite high prevalence, so far, there are no approved therapies for NASH. This progressive form of NAFLD has proven challenging to reverse with pharmacotherapies to date. On the other hand, preventing the progression from simple steatosis to steatohepatitis and fibrosis represents an attractive therapeutic target. Thus, there is a high unmet medical need to develop new effective therapeutic modalities targeting these key NASH mechanisms. Stool transplantation has been shown to ameliorate aspects related to NASH in pre-clinical models, but it does not offer a long-term solution to patients. The use of microbial consortia of defined composition may therefore represent a more patient-friendly approach at an early stage of disease. The role of the gut microbiome in NAFLD and other metabolic diseases is undisputable [2,4,42]. Intestinal microbial dysbiosis has been shown to contribute to the onset and progression of disease. Therefore, restoring eubiosis is of interest. In the current study, a specific consortium of nine intestinal commensal strains was tested in two in vivo models of NASH and shown to improve symptomatology and to prevent progression to fibrosis. This study convincingly showed that live biotherapeutic products (LBP) are a patient-friendly approach worth considering for development as therapy for NASH.

## Figures and Tables

**Table 1:** Biochemical characteristics after 2 weeks of intervention in the rat NASH model of PHT. HFGFD-VEH: group of NASH rats receiving sham gavage (vehicle); HFGFD-CON: group of NASH rats receiving the 9-strain bacterial consortium daily; HFGFD-FMT: group of NASH rats receiving fecal microbiota transplantation from control lean rats (1× transplantation followed by sham gavage).

	HFGFD-VEH *n* = 13	HFGFD-CON *n* = 11	HFGFD-FMT *n* = 11
Body weight pre-HFGFD (g)	254.46 ± 2.59	256 ± 2.1	256 ± 3.32
Body weight pre-trt. (g)	531.7 ± 10.3	543.6 ± 10.29	515.1 ± 13.48
Body weight gain with trt. (g)	22.08 ± 3.18	2.91 ± 3.55 *	−5 ± 3.47 *
Body weight post-trt. (g)	552.33 ± 11.77	546.6 ± 11.53	507 ± 12.39 *
Glucose (mg/dL)	182.45 ± 14.94	166.9 ± 14.89	171.36 ± 10.47
Insulin (ng/mL)	15.36 ± 3.6	9.24 ± 1.17	11.21 ± 1.13
HOMA-IR	7.89 ± 2.3	3.7 ± 0.54	4.68 ± 0.45
Albumin (g/dL)	2.9 ± 0.04	3.03 ± 0.05	3.13 ± 0.06*
Bilirubin (mg/dL)	0.09 ± 0.01	0.09 ± 0.02	0.11 ± 0.01
AST (IU/L)	205.56 ± 34.05	234.29 ± 45.72	172 ± 63.52
ALT (IU/L)	66.15 ± 4.36	67.1 ± 9.29	47.5 ± 31.31 *
TG (mg/dL)	27.92 ± 1.35	33 ± 1.91 *	33.54 ± 2.96 *
Total cholesterol (mg/dL)	75.54 ± 4.75	86.78 ± 3.89	78.91 ± 2.99
Cholesterol HDL (mg/dL)	41.93 ± 2.43	46.78 ± 1.97	45.54 ± 2.48
Cholesterol LDL (mg/dL)	25.27 ± 2.52	34.75 ± 3.4	27 ± 1.96

* *p* ≤ 0.05 versus HFGFD-VEH. Abbreviations: ALT, alanine aminotransferase; AST, aspartate aminotransferase; HDL, high-density lipoprotein; HFGFD, high-fat, high-glucose/fructose diet; HOMA-IR, Homeostasis model of insulin resistance index; LDL, low-density lipoprotein; TG, triglycerides.

**Table 2:** Hemodynamic measurements after 2 weeks of intervention in the rat NASH model of PHT. HFGFD-VEH: group of NASH rats receiving sham gavage (vehicle); HFGFD-CON: group of NASH rats receiving the 9-strain bacterial consortium daily; HFGFD-FMT: group of NASH rats receiving fecal microbiota transplantation from control lean rats (1× transplantation followed by sham gavage).

	HFGFD-VEH *n* = 13	HFGFD-CON *n* = 11	HFGFD-FMT *n* = 11
MAP (mmHg)	113.05 ± 4.53	119.95 ± 4.21	117.34 ± 5.14
PP (mmHg)	10.32 ± 0.22	9.58 ± 0.19 *	9.21 ± 0.12 *
SMABF (mL/[min × 100 g])	2.73 ± 0.24	2.71 ± 0.21	2.82 ± 0.17
SMAR (mmHg/mL × min × 100 g)	40.78 ± 3.25	40.79 ± 4.36	39.35 ± 2.36
IHVR (mmHg/mL × min × 100 g)	7.58 ± 1.27	4.20 ± 0.35 *	3.71 ± 0.21 *

* *p* ≤ 0.05 versus HFGFD-VEH. Abbreviations: HFGFD, high-fat, high-glucose/fructose diet; IHVR, intrahepatic vascular resistance; MAP, mean arterial pressure; PP, portal pressure; SMABF, superior mesenteric artery blood flow; SMAR, superior mesenteric artery resistance.

**Table 3:** Modulation of the top 1000 fatty liver disease genes in the liver of NASH rats. VEH: group of NASH rats receiving sham gavage (vehicle); CON: group of NASH rats receiving the 9-strain bacterial consortium daily; FMT: group of NASH rats receiving fecal microbiota transplantation from control lean rats (1× transplantation followed by sham gavage).

Rat Gene	Human Ortholog	CON/VEH Log2FC	CON/VEH pFDR	FMT/VEH Log2FC	FMT/VEH pFDR	Protein
Hspa1b	HSPA1B, HSPA1A	−3.48	0.002	−4.36	0.000	Heat shock 70 kDa protein 1B, and 1A
AABR07048992.1	HSPA8	−1.49	0.000	−1.50	0.000	Heat shock cognate 71 kDa protein
Hsph1	HSPH1	−0.74	0.060	−0.93	0.005	Heat shock protein 105 kDa
Pir	PIR	−0.74	0.084	-	-	Pirin
Sdf2l1	SDF2L1	−0.71	0.066	-	-	Stromal cell-derived factor 2-like protein 1
Dnajb9	DNAJB9	−0.71	0.016	−0.69	0.012	DNAJ homolog subfamily B member 9
LOC680121	HSPA8	−0.64	0.003	−0.48	0.024	Heat shock cognate 71 kDa protein
AABR07012795.1	PRDX1	−0.62	0.067	-	-	Peroxiredoxin−1
Hspa5	HSPA5	−0.61	0.058	−0.62	0.031	Endoplasmic reticulum chaperone BiP
Sult2a1	SULT2A1	−0.55	0.073	-	-	Sulfotransferase 2A1
Dusp6	DUSP6	−0.51	0.084	-	-	Dual specificity protein phosphatase 6
Calr	CALR	−0.40	0.030	−0.38	0.022	Calreticulin
Pnrc1	PNRC1	−0.40	0.081	-	-	Proline-rich nuclear receptor coactivator 1
Cdc42	CDC42	−0.36	0.000	−0.21	0.013	Cell division control protein 42 homolog
NEWGENE_620180	SLC40A1	−0.35	0.021	−0.43	0.002	Solute carrier family 40 member 1
Prdx1	PRDX1	−0.35	0.001	-	-	Peroxiredoxin-1
Litaf	LITAF	−0.34	0.000	-	-	Lipopolysaccharide-induced tumor necrosis factor-alpha factor
Atp2a2	ATP2A2	−0.32	0.065	−0.40	0.007	Sarcoplasmic/endoplasmic reticulum calcium ATPase 2
Slc10a1	SLC10A1	−0.30	0.081	−0.28	0.074	Sodium/bile acid cotransporter
Pdia3	PDIA3	−0.29	0.084	−0.29	0.051	Protein disulfide isomerase A3
Idh1	IDH1	−0.28	0.050	−0.32	0.008	Isocitrate dehydrogenase
Slc39a8	SLC39A8	−0.27	0.017	−0.19	0.093	Metal cation symporter ZIP8
Apoe	APOE	−0.26	0.067	−0.23	0.067	Apolipoprotein E
Maoa	MAOA	−0.25	0.049	−0.24	0.042	Amine oxidase
Ctsb	CTSB	−0.25	0.048	−0.34	0.001	Cathepsin B
Psmb4	PSMB4	−0.24	0.040	-	-	Proteasome subunit beta type-4
Ccnd3	CCND3	−0.24	0.051	-	-	G1/S-specific cyclin-D3
Dpp4	DPP4	−0.23	0.050	−0.35	0.000	Dipeptidyl peptidase 4
Aldoa	ALDOA	−0.23	0.067	-	-	Fructose bisphosphate aldolase A
Ctsd	CTSD	−0.22	0.069	−0.29	0.005	Cathepsin D
Rac1	RAC1	−0.21	0.000	−0.24	0.000	Ras-related C3 botulinum toxin substrate 1
Enpp1	ENPP1	−0.20	0.057	−0.26	0.004	Ectonucleotide pyrophosphatase/phosphodiesterase 1
Calm1	CALM1	−0.19	0.064	-	-	Calmodulin−1
Sdc2	SDC2	−0.19	0.064	−0.33	0.000	Syndecan-2
Akt1	AKT1	0.20	0.056	-	-	RAC-alpha serine/threonine-protein kinase
Abcg3l3	ABCG2	0.36	0.078	-	-	Broad substrate specificity ATP-binding cassette transporter ABCG2
Atm	ATM	0.39	0.006	-	-	Serine-protein kinase ATM
Abcg3l1	ABCG2	0.42	0.019	-	-	Broad substrate specificity ATP-binding cassette transporter ABCG2
Inhba	INHBA	0.48	0.099	-	-	Inhibin beta A chain
Abcc5	ABCC5	0.49	0.097	-	-	ATP-binding cassette sub-family C member 5
VEGFA	VEGFA	0.57	0.003	0.50	0.007	Vascular endothelial growth factor A
Egf	EGF	0.64	0.030	0.56	0.042	Pro-epidermal growth factor
LOC108348190	EGF	0.72	0.046	-	-	Pro-epidermal growth factor
Thrsp	THRSP	1.07	0.049	0.93	0.067	Thyroid hormone-inducible hepatic protein

**Table 4:** Putatively downregulated (as indicated by a downward arrow) pro-fibrotic signaling pathways in the liver of NASH rats treated with the 9-strain consortium.

Pathway ID	N	q-Value	Human Orthologs	Description
WP3888↓	21	0.090	RPS11, TPP1, ALDOA, PPP1CA, CTNND1, SDCBP, DNAJA1, CDC42, SSR4, DNAJB9, RAP1B, RHOA, PFN1, YWHAE, PSMD11, PAK2, SDF2L1, CALR, ATP6V0D1, HSPA1A, RAC1	VEGFA-VEGFR2 Signaling pathway
WP4656↑	8	0.067	CSPP1, CEP104, DVL1, OFD1, ATM, BBS4, PCM1, CEP120	Joubert Syndrome
WP428↓	7	0.091	RHOA, RAC1, WNT11, CCND3, PPP3R1, GPC4, NLK	Wnt Signaling
P00016↓	6	0.031	ARPC4, CDC42, ARPC5, PFN1, PAK2, RAC1	Cytoskeletal regulation by Rho GTPase
pid_21478↓	5	0.007	ARPC4, CDC42, PIR, ACTR3, RAC1	y branching of actin filaments
pid_5967↓	5	0.007	ARPC4, CDC42, RHOA, ACTR3, RAC1	Role of pi3k subunit p85 in regulation of actin organization and cell migration
R-HSA-390450↓	3	0.030	TCP1, CCT4, CCT8	Folding of actin by CCT/TriC
R-HSA-5625970↓	3	0.030	CDC42, RHOA, RAC1	RHO GTPases activate KTN1
R-HSA-389960↓	3	0.053	TCP1, CCT4, CCT8	Formation of tubulin folding intermediates by CCT/TriC

**Table 5:** Biochemical characteristics after 4 weeks of intervention in the STAM™ mouse study. CD + VEH: group of control diet mice receiving sham gavage (vehicle); STAM + VEH: group of STAM mice receiving sham gavage (vehicle); STAM + CON: group of STAM mice receiving the 9-strain bacterial consortium daily; STAM + TLM: group of STAM mice receiving Telmisartan daily.

	CD + VEH *n* = 8 9 Weeks	STAM + VEH *n* = 8 9 Weeks	STAM + CON *n* = 10 9 Weeks	STAM + TLM *n* = 8 9 Weeks	STAM + VEH *n* = 8 12 Weeks	STAM + CON *n* = 8 12 Weeks
ALT (IU/L)	24.00 *** ± 1.27	39.38 ± 2.41	43.5 ± 3.28	35.00 ± 2.39	65.13 ± 14.04	60.63 ±12.81
TG (mg/dL)	79.63 * ± 10.46	500.9 ± 152.5	679.9 ± 91.35	465.6 ± 107.6	926.4 ± 251.6	1017 ± 248.8
Total cholesterol (mg/dL)	76.92 **** ± 3.51	125.7 ± 8.11	124.8 ± 4.32	132.7 ± 2.71	187.3 ± 41.99	186.9 ± 38.81
Cholesterol HDL (mg/dL)	57.84 ** ± 2.75	83.69 ± 7.13	75.55 ± 3.31	97.21 ± 4.73	72.24 ± 7.46	70.21 ± 6.84
Cholesterol LDL (mg/dL)	13.1 ± 0.66	14.68 ± 1.20	13.64 ± 0.74	18.78 * ± 1.10	24.35 ± 6.12	23.76 ± 5.16

*, **, ***, and **** *p* ≤ 0.05, ≤0.01, ≤0.001, and ≤0.0001, respectively, versus STAM + VEH. Abbreviations: ALT, alanine aminotransferase; HDL, high-density lipoprotein; LDL, low-density lipoprotein; TG, triglycerides.

## Data Availability

Data is contained within the article.

## Supplementary Materials

The following supporting information can be downloaded at: https://www.mdpi.com/article/10.3390/biomedicines10051191/s1. Figure S1 depicts the body weight gain and insulin resistance in NASH rats (fasting insulin levels and HOMA-IR); Figure S2 displays the histological evaluation of the H&E-stained liver sections collected from the NASH rats in bar diagrams; Figure S3 shows representative images of liver parenchyma stained with H&E or liver fibrosis stained with Sirius red in sections collected from the NASH rats; Figure S4 shows the results on the microbial taxonomic composition in the cecum of NASH rats; Figure S5 displays the results obtained on the hepatic gene expression in NASH rats; Figure S6 shows the whole blood HbA1c measured in the STAM™ mice; Figure S7 shows representative images of H&E-stained liver sections collected from the STAM™ study; Figure S8 shows representative images of Sirius red-stained, and F4/80 immunostained liver sections collected from the STAM™ study; Figure S9 displays the immunohistochemistry of fibronectin (bar plot), and the representative images of fibronectin immunostained liver sections collected from the STAM™ study at 12 weeks of age [43,44,45,46,47,48,49,50,51,52,53,54,55].
