# Supplementary material for: A Nine-Strain Bacterial Consortium Improves Portal Hypertension and Insulin Signaling and Delays NAFLD Progression In Vivo
Source: Biomedicines. 2022 May 20;10(5):1191. doi: 10.3390/biomedicines10051191 (PMC9175091; doi:10.3390/biomedicines10051191)
Supplement: Supplementary file 1 [file biomedicines-10-01191-s001.zip › Pinheiro et al_Supplementary Materials.pdf]

biomedicines

Supplementary Materials:

Disease induction on the rat NASH model of PHT

To induce the NASH phenotype, rats had *ad libitum* access to a high-fat high-glucose/fructose diet (HFGFD) consisting of 30% fat (butter, coconut oil, palm oil, beef tallow) with mainly saturated fatty acids (5.73kcal/g; Ssniff Spezialdiäten GmbH, Soest, Germany), supplemented with cholesterol (1g/kg), and a beverage of high glucose/fructose content (42g/L, 45% glucose and 55% fructose) providing 157.7kcal/L.

Immunohistochemistry of liver samples on the STAM™ mouse model of NASH

In the STAM™ study, mouse liver specimens were stored at -80°C embedded in Optimal Cutting Temperature (O.C.T., Sakura Finetek Japan, Japan) compound for immunohistochemistry and fixed in acetone. O.C.T.-embedded specimens were used for F4/80 and fibronectin immunohistochemistry. First, endogenous peroxidase activity was blocked using 0.03% H_2O_2 for 5 minutes, followed by incubation with Block Ace (Dainippon Sumitomo Pharma Co. Ltd., Japan) for 10 minutes. Then, sections were incubated with anti-F4/80 (Monoclonal Antibody, T-2006, BMA Biomedicals), or anti-fibronectin antibody (ab2413, Abcam) overnight at 4°C. After incubation with secondary antibody (Vector Laboratories), enzyme-substrate reactions were performed using 3,3'-diaminobenzidine/H_2O_2 solution (Nichirei Bioscience Inc., Japan). Bright field images of immunostained sections were captured around the central vein using a digital camera (DFC295; Leica, Germany) at 200-fold magnification.

Cecal whole metagenome shotgun sequencing on the NASH rats: DNA Extraction, Library Preparation, taxonomic profiling, and pathway analyses

Genomic DNA was extracted from approx. 0.2g of cecum content of each rat using the ZymobioMics DNA Miniprep Kit (Zymo Research, Irvine, CA, USA) according to manufacturer's instructions and quantified using the Qubit™ Flex Fluorometer (Thermo Fisher Scientific, Wilmington, DE, USA) and used for whole metagenome shotgun (WMS) sequencing. WMS sequencing, taxonomic- and pathway profiling were performed by CosmosID (Rockville, MD, USA). As result, 2x150bp Illumina reads were mapped to a proprietary microbial genome database for taxonomic profiling [43] and to UniRef90 and MetaCyc for pathway profiling [44]. Treatment-induced microbial composition modulation was assessed with both unsupervised analysis (multi-dimensional scaling using UniFrac distances), and supervised analysis (sparse partial least-squares discriminant analysis, sPLS-DA), using the R packages phyloseq [45] and mixOmics [46], respectively. Classifier performance for the latter was appreciated through the balanced error rate (BER): BER=0.5×(FP/(TN+FP)+FN/(TP+FN)). Microbiome features, comprising diversity estimates and inferred metabolite biosynthesis capacity were treated as non-normally distributed and non-parametric Wilcoxon signed-rank testing was performed to compute statistical significance of group comparison.

Liver transcriptomics on the NASH rats: extraction, RNA Library Preparation, and HiSeq Sequencing analyses

RNA extraction, RNA library preparations, and sequencing reactions were conducted at GENEWIZ, LLC. (South Plainfield, NJ, USA) as follows: total RNA was extracted from fresh frozen liver tissue samples using the Qiagen RNeasy Plus Universal mini kit following manufacturer's instructions (Qiagen, Hilden, Germany). RNA samples were quantified using Qubit 2.0 Fluorometer (Life Technologies, Carlsbad, CA, USA), and RNA integrity was measured using the RNA Screen Tape on Agilent 2200 TapeStation (Agilent Technologies, Palo Alto, CA, USA). RNA sequencing libraries were prepared using TruSeq Stranded mRNA library prep kit following manufacturer's protocol (Illumina, Cat# RS-122-2101). First, mRNA's were enriched with Oligo d(T) beads. Enriched mRNA's were fragmented for 8 minutes at 94°C. Subsequently, first strand and second strand cDNA were synthesized. The second strand of cDNA was marked by incorporating dUTP's during synthesis. cDNA fragments were adenylated at 3'ends and indexed adapters were ligated to cDNA fragments. Limited cycle PCR was used for library enrichment. The incorporated dUTP's in second strand cDNA quenched the amplification thus preserving strand specificity. Sequencing libraries were validated using DNA Analysis Screen Tape on the Agilent 2200 TapeStation (Agilent Technologies, Palo Alto, CA, USA), and quantified by using Qubit 2.0 Fluorometer (Invitrogen, Carlsbad, CA) as well as by quantitative PCR (KAPA Biosystems, Wilmington, MA, USA). The pooled libraries were clustered on 7 lanes of a flowcell. After clustering, the flowcell was loaded on the Illumina HiSeq instrument (4000 or equivalent) according to manufacturer's instructions and sequenced using a 2x150bp Paired End (PE) configuration. Image analysis and base calling were conducted by the HiSeq Control Software (HCS). Raw sequence data (.bcl files) generated from Illumina HiSeq was converted into fastq files and de-multiplexed using Illumina's bcl2fastq 2.17 software. One mismatch was allowed for index sequence identification.

Strand-specific gene expression profiling was achieved through mapping of Illumina 2x150bp reads to the Ensembl *Rattus norvegicus* Rnor_6.0 transcriptome and processing the alignments with feature Counts from the subread package [47]. Rat Ensembl gene (version 104) IDs were mapped to human Ensembl gene equivalents and HUGO Gene Nomenclature Committee (HGNC) symbols through

Ensembl Biomart. Resulting uniquely mapping fragment counts were taken into differential gene expression analysis with the R package DESeq2 [48] to obtain differential expressed genes with Benjamini & Hochberg false discovery rate (FDR) adjusted P-values. Outlier analysis was performed with orthogonal partial least squares analysis using the R package ropls [49]. Simultaneously, sPLS-DA was performed to estimate variable importance (VIP). Liver genes discriminant for treatment *vs.* vehicle contrasts were retained as having a VIP>1 and FDR adjusted P-value<0.1. Functional over-representation analysis (ORA) in these genes was performed with the R package clusterProfiler [50], using the functional annotation database WikiPathways (WP) [51], MetaCyc (MC) [52], Reactome (RT) [53], and Biocarta (BC) [54]. The Harmonizome database [55] provides curated and ranked disease gene associations, including a Fatty Liver gene set of 11,463 genes. The top 1,000 fatty liver associated genes were used to select disease related genes from differential gene expression analysis.

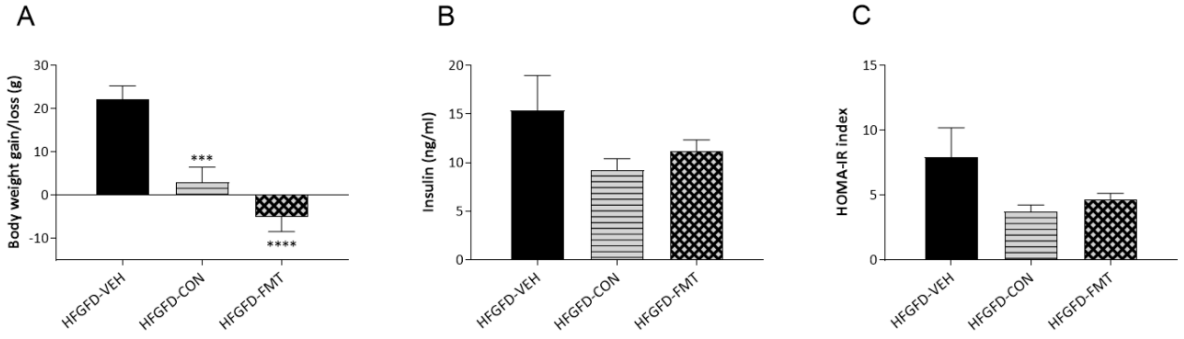

Figure S1. Body weight gain and evaluation of insulin resistance in NASH rats. (A) Body weight gain after 2 weeks of intervention. (B) Fasting insulin levels measured in the blood after 2 weeks of intervention. (C) Calculated Homeostatic Model Assessment for Insulin Resistance index. HFGFD-VEH: group of NASH rats receiving sham gavage (vehicle); HFGFD-CON: group of NASH rats receiving the 9-strain bacterial consortium daily; HFGFD-FMT: group of NASH rats receiving fecal microbiota transplantation from control lean rats (1x transplantation followed by sham gavage). ***,**** P≤0.001 and ≤0.0001, respectively, *versus* HFGFD-VEH.

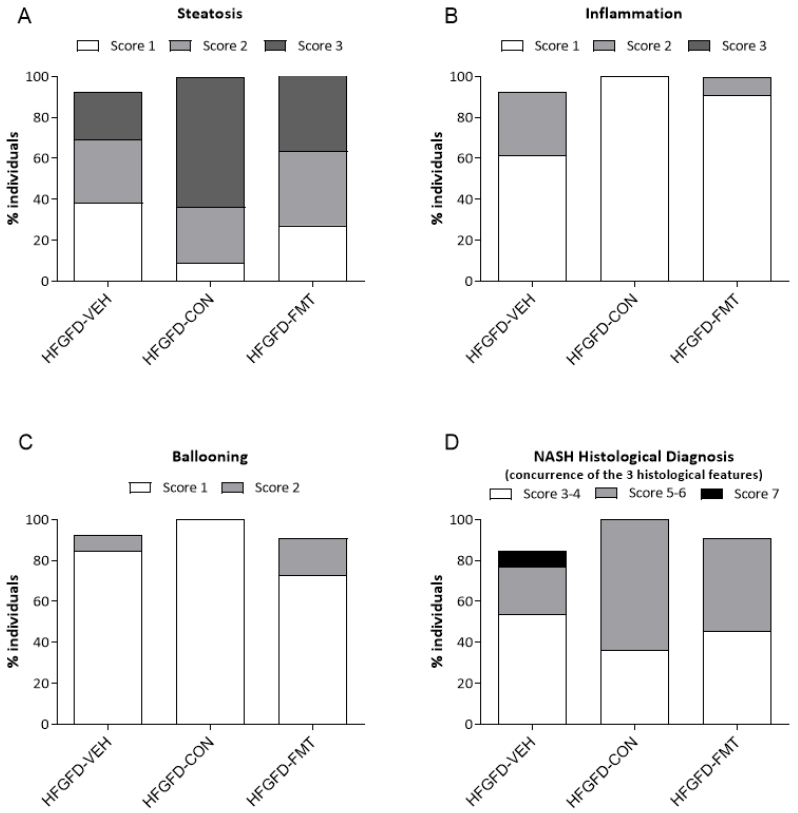

Figure S2. Histological evaluation of the H&E-stained liver sections collected from the NASH rats. Evaluation followed the NASH Clinical Research Network Histological Scoring System. The three first bar diagrams represent the percentage of individuals who present (A) steatosis, (B) inflammation, and (C) ballooning in the different groups. Each bar includes the percentage of individuals scored with 1 (white), 2 (light gray), or 3 (dark grey); the last bar diagram (D) represents the combined scores. HFGFD-VEH: group of NASH rats receiving sham gavage (vehicle); HFGFD-CON: group of NASH rats receiving the 9-strain bacterial consortium daily; HFGFD-FMT: group of NASH rats receiving fecal microbiota transplantation from control lean rats (1x transplantation followed by sham gavage).

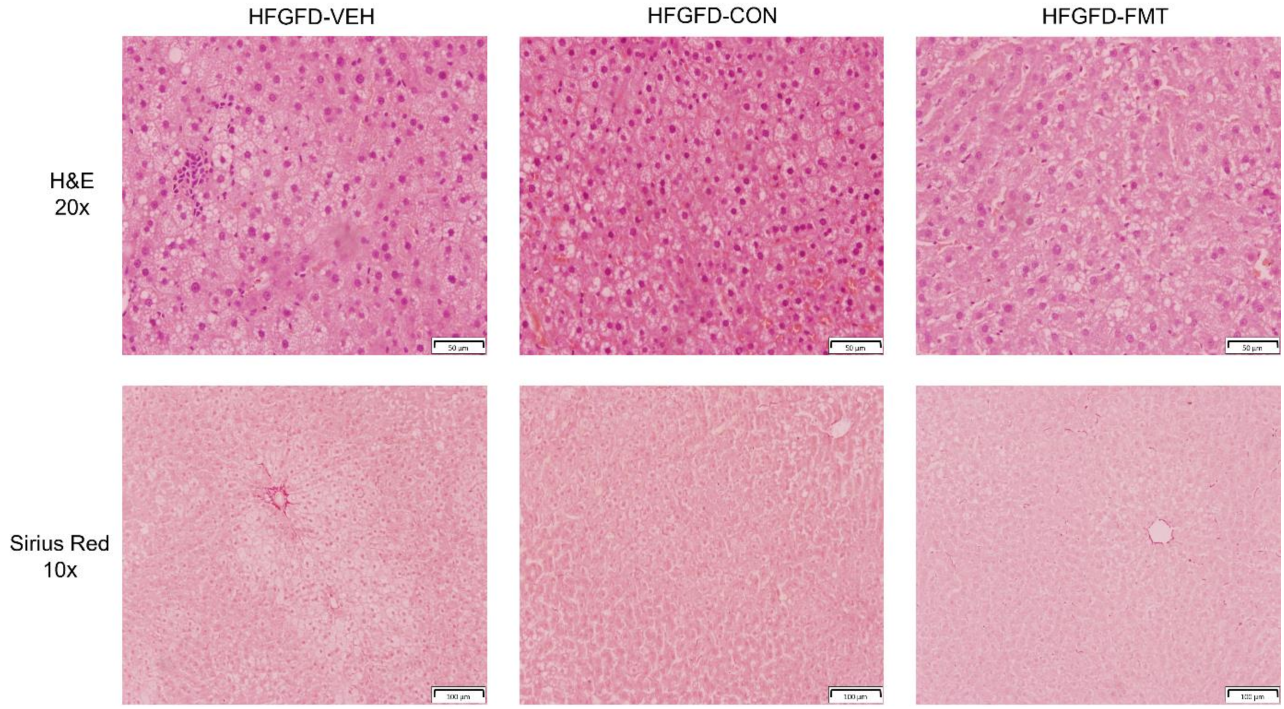

Figure S3. Representative images showing liver parenchyma stained with hematoxylin and eosin (H&E, 20x) or liver fibrosis stained with Sirius red (10x) in sections collected from the NASH rats. HFGFD-VEH: group of NASH rats receiving sham gavage (vehicle); HFGFD-CON: group of NASH rats receiving the 9-strain bacterial consortium daily; HFGFD-FMT: group of NASH rats receiving fecal microbiota transplantation from control lean rats (1x transplantation followed by sham gavage).

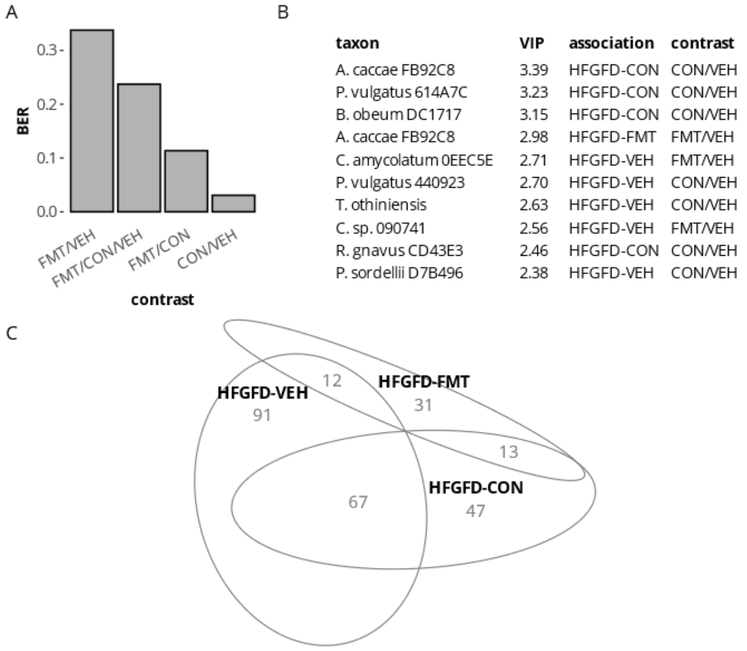

Figure S4. Microbial taxonomic composition in the cecum of NASH rats (sPLS-DA classifier). (A) Per-contrast balanced error rate (BER). (B) Top 10 HFGFD-VEH discriminant taxa according to classifier estimated variable importance (VIP). (C) Euler diagram of contrast specific taxa. HFGFD-VEH: group of NASH rats receiving sham gavage (vehicle); HFGFD-CON: group of NASH rats receiving the 9-strain bacterial consortium daily; HFGFD-FMT: group of NASH rats receiving fecal microbiota transplantation from control lean rats (1x transplantation followed by sham gavage).

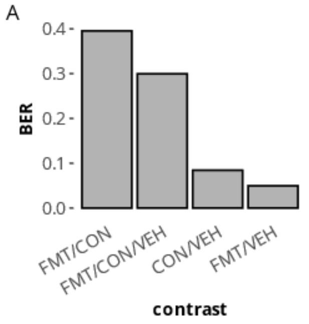

B

gene	VIP	association	contrast	log2FC
Nf2	33.0	HFGFD-CON	CON/VEH	0.37
Anapc16	29.3	HFGFD-FMT	FMT/VEH	0.78
Sec62	29.0	HFGFD-VEH	FMT/VEH	-0.38
Bcl2l2	25.7	HFGFD-CON	CON/VEH	0.46
Mprip	25.5	HFGFD-CON	CON/VEH	0.34
Arfip2	25.4	HFGFD-CON	CON/VEH	1.05
Crcp	25.4	HFGFD-FMT	FMT/VEH	0.57
Bcl2l2	25.3	HFGFD-FMT	FMT/VEH	0.45
LOC103694404	24.6	HFGFD-VEH	FMT/VEH	-1.28
AABR07048992.1	24.1	HFGFD-VEH	CON/VEH	-1.49

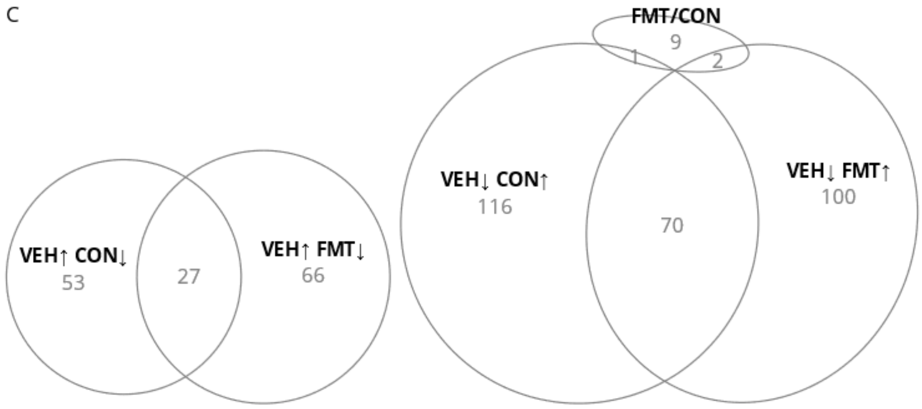

Figure S5. Liver gene expression in NASH rats (sPLS-DA classifier). (A) Per-contrast balanced error rate (BER). (B) Top 10 discriminant genes. (C) Euler diagram of the 444 contrast specific genes. HFGFD-VEH: group of NASH rats receiving sham gavage (vehicle); HFGFD-CON: group of NASH rats receiving the 9-strain bacterial consortium daily; HFGFD-FMT: group of NASH rats receiving fecal microbiota transplantation from control lean rats (1x transplantation followed by sham gavage).

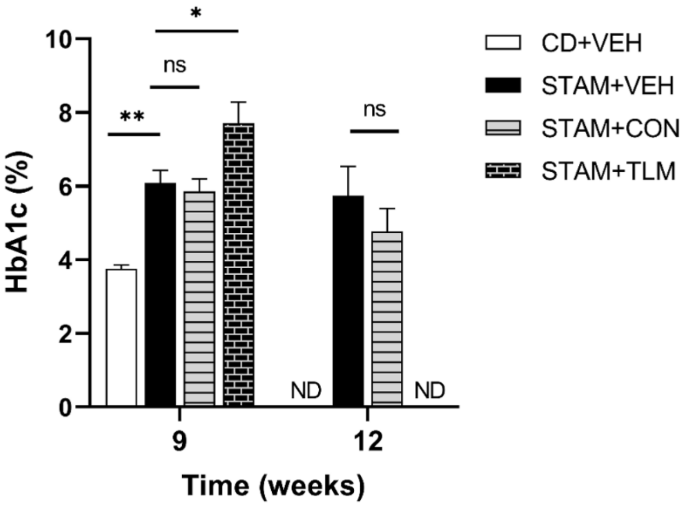

Figure S6. Whole blood HbA1c (%) measured in the STAM™ study**.** Whole blood was collected at 9 and 12 weeks of age (euthanasia 1 and 2, respectively) in heparinized tubes and used for analysis of glycated hemoglobin, a marker that indicates the presence of excessive glucose in the bloodstream. CD+VEH: group of control diet mice receiving sham gavage (vehicle); STAM +VEH: group of STAM mice receiving sham gavage (vehicle); STAM+CON: group of STAM mice receiving the 9-strain bacterial consortium daily; STAM+TLM: group of STAM mice receiving Telmisartan daily. ND: not determined for the CD+VEH and STAM +TLM groups at 12 weeks. ns: not significant. *,** P≤0.05 and ≤0.01, respectively, *versus* STAM+VEH.

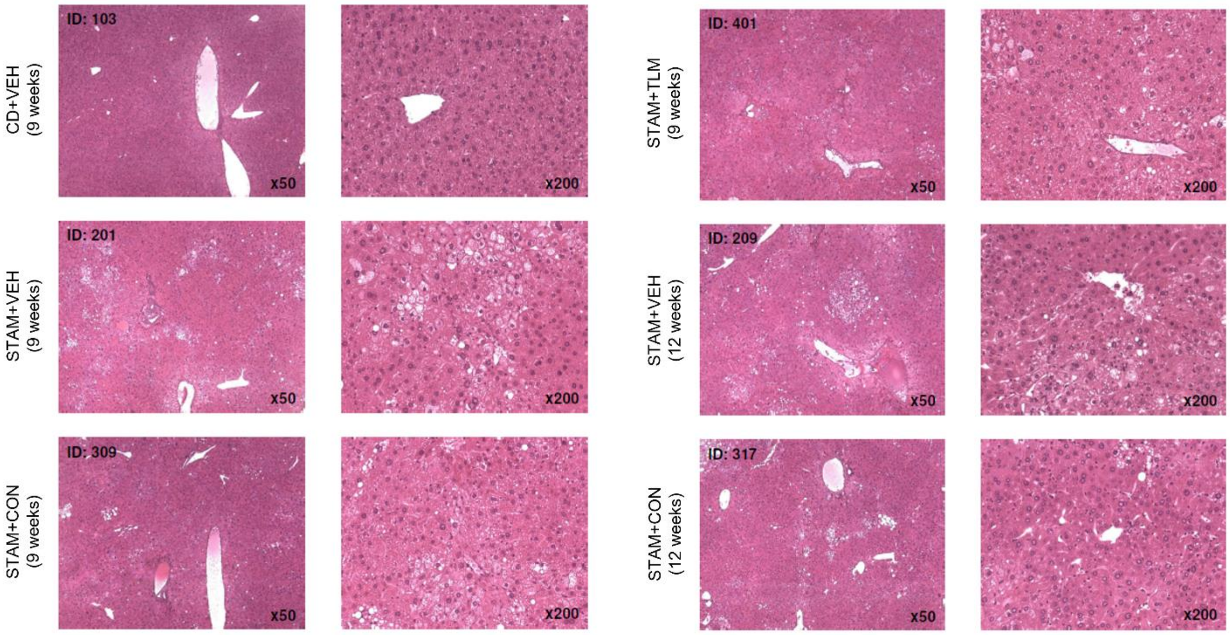

Figure S7. Representative images of H&E-stained liver sections collected from the STAM™ study. Liver sections were collected at both 9 and 12 weeks of age (euthanasia 1 and 2, respectively). CD+VEH: group of control diet mice receiving sham gavage (vehicle); STAM+VEH: group of STAM mice receiving sham gavage (vehicle); STAM+CON: group of STAM mice receiving the 9-strain bacterial consortium daily; STAM+TLM: group of STAM mice receiving Telmisartan daily.

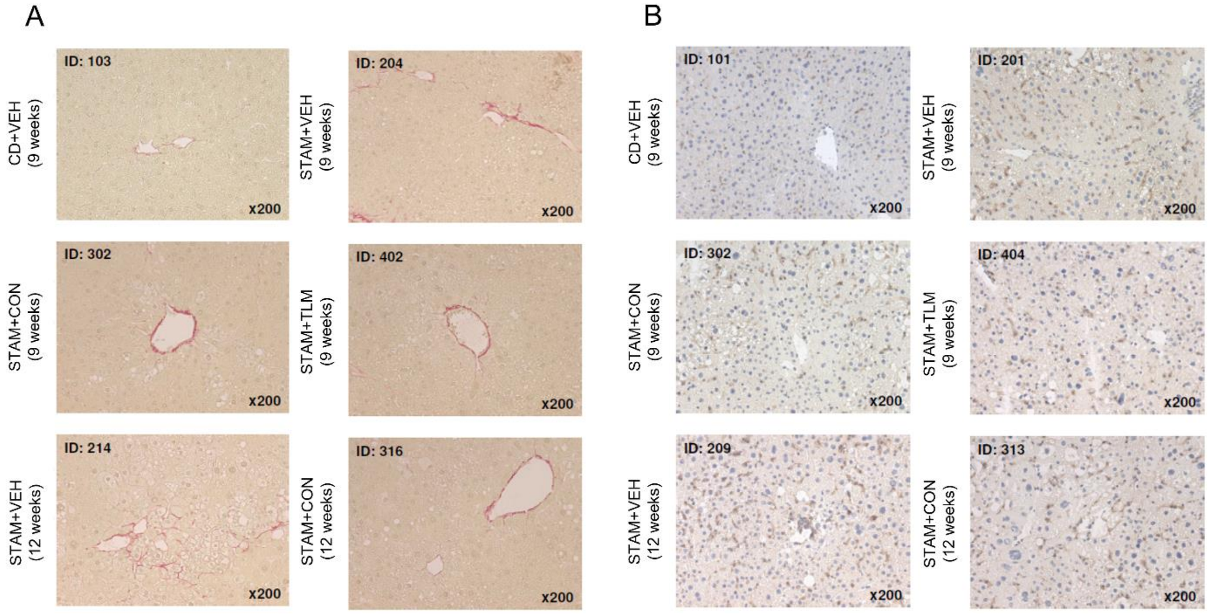

Figure S8. Representative images of (A) Sirius red-stained, and (B) F4/80 immunostained liver sections collected from the STAM™ study. Liver sections were collected at both 9 and 12 weeks of age (euthanasia 1 and 2, respectively). CD+VEH: group of control diet mice receiving sham gavage (vehicle); STAM+VEH: group of STAM mice receiving sham gavage (vehicle); STAM+CON: group of STAM mice receiving the 9-strain bacterial consortium daily; STAM+TLM: group of STAM mice receiving Telmisartan daily.

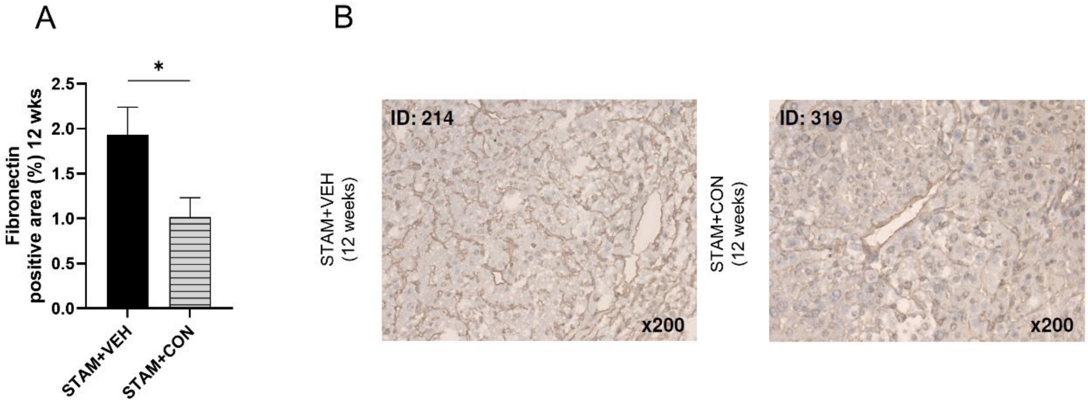

Figure S9. Immunohistochemistry of (A) Fibronectin at 12 weeks, and (B) representative images of fibronectin immunostained liver sections collected from the STAM™ study at 12 weeks of age (euthanasia 2). CD+VEH: group of control diet mice receiving sham gavage (vehicle); STAM+VEH: group of STAM mice receiving sham gavage (vehicle); STAM+CON: group of STAM mice receiving the 9-strain bacterial consortium daily; STAM+TLM: group of STAM mice receiving Telmisartan daily. * $P \leq 0.05$.
